# Duck gut viral metagenome analysis captures snapshot of viral diversity

**DOI:** 10.1186/s13099-016-0113-5

**Published:** 2016-06-09

**Authors:** Mohammed Fawaz, Periyasamy Vijayakumar, Anamika Mishra, Pradeep N. Gandhale, Rupam Dutta, Nitin M. Kamble, Shashi B. Sudhakar, Parimal Roychoudhary, Himanshu Kumar, Diwakar D. Kulkarni, Ashwin Ashok Raut

**Affiliations:** OIE Reference Laboratory for Avian Influenza, ICAR-National Institute of High Security Animal Diseases, Bhopal, Madhya Pradesh India; College of Veterinary Sciences and Animal Husbandry, Central Agricultural University, Aizawl, Mizoram India; Laboratory of Immunology, Department of Biological Sciences, Indian Institute of Science Education and Research, Bhopal, Madhya Pradesh India

**Keywords:** Duck, Virome, Viral metagenomics, Gut, *Papillomaviridae*, Next generation sequencing

## Abstract

**Background:**

Ducks (*Anas platyrhynchos*) an economically important waterfowl for meat, eggs and feathers; is also a natural reservoir for influenza A viruses. The emergence of novel viruses is attributed to the status of co-existence of multiple types and subtypes of viruses in the reservoir hosts. For effective prediction of future viral epidemic or pandemic an in-depth understanding of the virome status in the key reservoir species is highly essential.

**Methods:**

To obtain an unbiased measure of viral diversity in the enteric tract of ducks by viral metagenomic approach, we deep sequenced the viral nucleic acid extracted from cloacal swabs collected from the flock of 23 ducks which shared the water bodies with wild migratory birds.

**Result:**

In total 7,455,180 reads with average length of 146 bases were generated of which 7,354,300 reads were *de novo* assembled into 24,945 contigs with an average length of 220 bases and the remaining 100,880 reads were singletons. The duck virome were identified by sequence similarity comparisons of contigs and singletons (BLASTx E score, <10^−3^) against viral reference database. Numerous duck virome sequences were homologous to the animal virus of the *Papillomaviridae* family; and phages of the *Caudovirales*, *Inoviridae*, *Tectiviridae*, *Microviridae* families and unclassified phages. Further, several duck virome sequences had homologous with the insect viruses of the *Poxviridae*, *Alphatetraviridae*, *Baculoviridae, Densovirinae, Iflaviridae* and *Dicistroviridae* families; and plant viruses of the *Secoviridae, Virgaviridae, Tombusviridae* and *Partitiviridae* families, which reflects the diet and habitation of ducks.

**Conclusion:**

This study increases our understanding of the viral diversity and expands the knowledge about the spectrum of viruses harboured in the enteric tract of ducks.

## Background

The duck (*Anas platyrhynchos*) is one of the economically important poultry species as a source of meat, eggs and feathers [1]. Ducks harbour most of the hemagglutinin (HA) and neuraminidase (NA) subtypes of avian influenza viruses that are currently known [2, 3] and serve as the principal natural reservoir host for influenza A viruses [2, 4–6]. Influenza A viruses maintained in wild aquatic birds have been associated with stable host switch events to novel hosts including mammals and domestic gallinaceous poultry leading to the emergence of novel influenza A viruses [7, 8].

To control the outbreaks of emerging or re-emerging viral diseases and prevent the transmission of viruses from the reservoir host, monitoring the virome status in the reservoir hosts is essential [9]. Further, understanding the viral diversity in the poultry gut will improve the knowledge of enteric disease syndromes and the feed conversion efficiency of the poultry species [10]. In recent years, next generation sequencing technology based viral metagenomics has provided a powerful tool for large-scale detection of known and unknown viruses existing in the reservoir host [11, 12]. Using this approach, known and novel viruses have been characterized from the enteric tract of turkey [10], bats [13], pigs [14], rodents [15], pigeon [16], ducks [17] and ferrets [18]. To obtain an unbiased measure of the viral diversity in the enteric tract of ducks, we deep sequenced viral nucleic acid isolated from cloacal swabs of 23 ducks collected from Bhoj wetland of Bhopal, the capital of the central Indian state of Madhya Pradesh. The present study revealed that the duck gut virome contained sequences related to a wide range of animal, insect, plant, and bacterial viruses. This study increases our understanding of the viral diversity present in the enteric tract of ducks. Further, this virome dataset provide a baseline faecal virome of the ducks and will be used as reference for identification of future changes in its virome composition, which may be associated with disease outbreaks or environmental changes.

## Methods

### Ethics statement

The experiments were approved by the institutional animal ethics committee of ICAR—National Institute of High Security Animal Diseases (Approval no. 81/IAEC/HSADL/13) and performed under the guidance of the Committee for the Purpose of Control and Supervision of Experiments on Animals (CPCSEA), Ministry of Environment and Forests, Govt. of India.

### Sample collection and processing

The Bhoj wetland consists of two lakes the Bhojtal and the lower lake, located in the city of Bhopal, the capital of the central Indian state of Madhya Pradesh. The lakes are home to a diverse flora and fauna, including many water birds. This wetland has been designated as wetland of international importance under the international Ramsar convention. The ducks and geese in the lake share water bodies with wild migratory birds. Cloacal swabs were collected from the flock of 23 ducks and the swabs were placed in the eppendorf tubes containing 200 µl Hank’s Balanced Salt Solution (HBSS). The eppendorf tubes were vortexed and centrifuged at 6000*g* for 10 s. The swabs were once again washed with 200 µl HBSS at 7400 g for 15 s. The total volume of washings were pooled and passed through 0.45 µm filter. The filtrate was ultra centrifuged at 37,000 rpm for 4 h at 4 °C. The supernatant was discarded and the pellet was resuspended in 50 µl HBSS for overnight softening, then resuspended in 200 µl HBSS and stored at −80 °C.

### Extraction of viral nucleic acids and next generation sequencing

The filtrate was treated with a cocktail of nucleases (TURBO DNase, Benzonase, RNase I) at 37 °C in dry bath for 2 h. Viral nucleic acid was extracted from the nuclease treated filtrate using High Pure Viral Nucleic acid Kit (Roche) and QIAamp viral RNA mini kit (Qiagen). NanoDrop spectrophotometer was used for quantification of viral nucleic acids. The viral nucleic acids were reverse transcribed using SuperScript III reverse transcriptase (Invitrogen) as per manufacturer’s protocol with random hexamer primer. Further, double strand cDNA was synthesized using SMARTer Ultra Low input RNA kit. The paired-end library was prepared using Illumina TruSeq RNA Library Preparation v2 Kit. Fragmentation of DNA was done using Covaris S2 shearing that generated double-stranded DNA fragments comprising of 3′ or 5′ overhangs. Further, the fragmented DNA was end-repaired followed by A-tail adapter ligation. Selective enrichment of adapter ligated DNA fragments was done through PCR amplification and validation of library was done on Bioanalyzer 2100 using HS DNA Chip. The DNA library was sequenced for paired end 2 × 150 sequences on Illumina MiSeq platform.

### Bioinformatics

The paired end reads were trimmed of their set primer sequences and the sequences were then *de novo* assembled into contigs using CLC genomics workbench software, with criteria of at least 95 % identity over 35-bp to merge two fragments and minimum contig length of 100 bases. The assembled contigs and singleton sequences greater than 100 bp were compared to the NCBI GenBank nonredundant nucleotide and reference viral protein databases (http://www.ncbi.nlm.nih.gov/refseq/) using BLASTn and BLASTx, respectively. On the basis of the best BLASTx result, sequences were classified into their likely taxonomic groups of origin based on the best-hit (lowest E score) sequence match. An E value of <10^−3^ was the cut-off value for significant hits.

### Phylogenetic analysis

All alignments and phylogenetic analyses were based on the translated amino acid sequences. Putative open reading frames (ORFs) in the genome were predicted by NCBI ORF finder and BLASTn and BLASTp were performed to determine identity. Phylogenetic analyses were performed using duck viral sequences, their best BLASTp hits, and representative members of related viral species or genera. Sequence alignment was performed using CLUSTALW with the default settings [19]. Aligned sequences were trimmed to match the genomic regions of the viral sequences obtained in the study. A phylogenetic tree with 1000 bootstrap resamples of the alignment data sets was generated using the neighbor-joining method based on the p-distance model in MEGA version 6.06 [20].

## Results and discussion

### Viral metagenomic overview

In this study 7,455,180 reads with an average length of 146 bases were generated and these sequences were *de novo* assembled into 24,945 contigs and 100,880 reads remained as singletons.

**Fig. 1:**
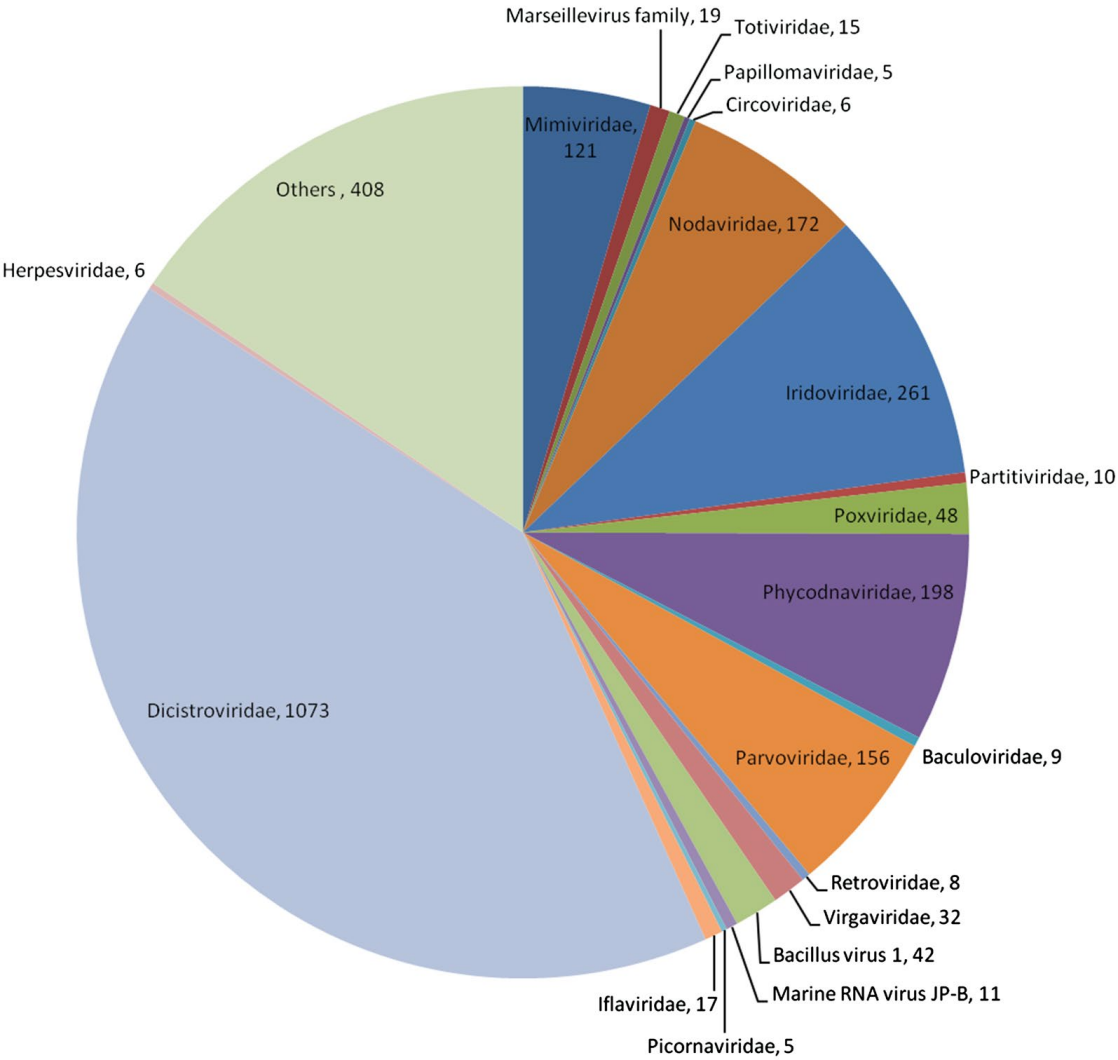
Taxonomic distributions of the eukaryotic virus-related sequences from duck gut virome. Assembled sequences were compared to the reference sequences in the viral protein database using BLASTx (E score, 10^−3^). The number of sequences with identities to eukaryotic viruses is shown

**Fig. 2:**
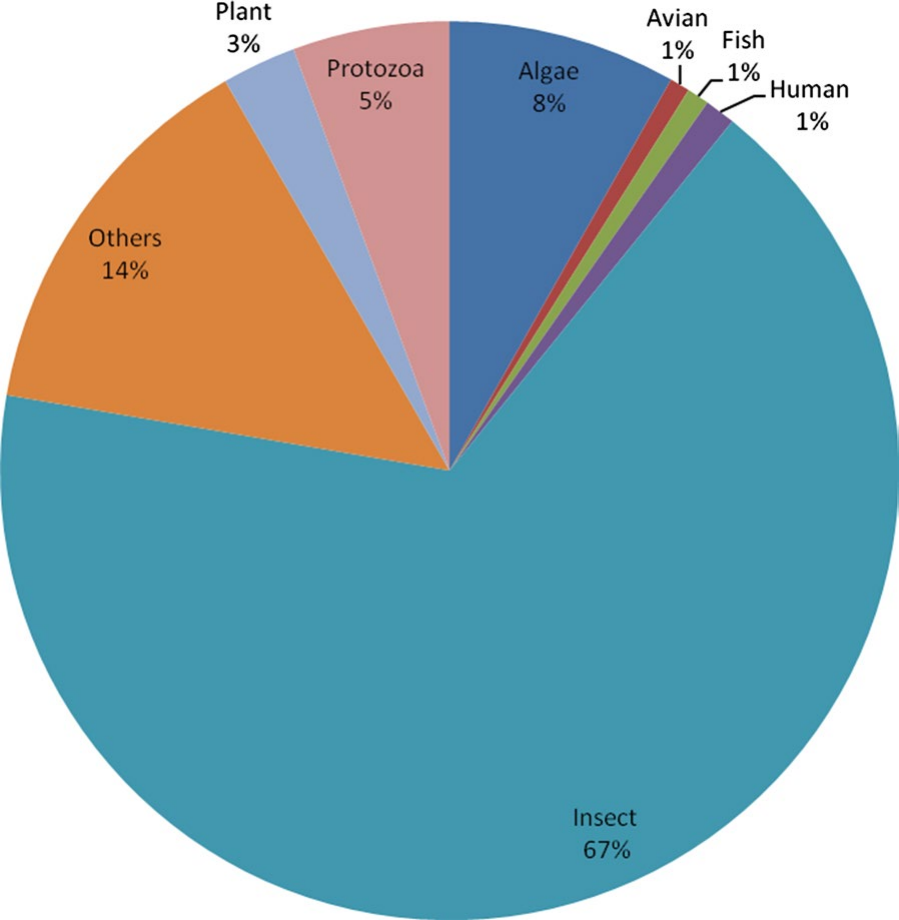
Host wise distribution of eukaryotic viruses catalogued in the duck gut virome

The average lengths of assembled contigs and singletons were 220 and 142 bases respectively. Both contigs and singletons longer than 100-bp were blasted against reference viral protein database, in all 11,340 (9.54 %) sequences had hits with viral protein sequences. The remaining large fraction of virome sequences were unclassifiable using BLASTx against the current viral database; it’s likely due to highly divergent nature of viral sequences from those of known prokaryotic and eukaryotic viral sequences [13, 21]. However, this fraction is consistent with the previous viral metagenomic studies [9, 10, 21–24]. Of the 9.54 % sequences, 7.33 % (8718) sequences had similarity to phages and 2.21 % (2622) sequences had similarity to the members of eukaryotic virus families (E value, <10^−3^), including *Mimiviridae, Partitiviridae, Poxviridae, Phycodnaviridae, Parvoviridae, Tombusviridae, Retroviridae, Dicistroviridae, Picornaviridae, Iflaviridae* covering viruses from invertebrates, vertebrates, and plants (Fig. 1). Further we did host wise classification of these eukaryotic viruses into insect, plant, human, avian, fish, algae and protozoa viruses (Fig. 2). Most of the reads were related to insect viruses, particularly members of the family *Dicistroviridae*.

### Insect, plant and phage viruses

Sequences relating to insect viruses comprised the largest proportion within the eukaryotic family viruses in duck gut virome (Fig. 2). The sequences had similarity to insect virus families, including *Dicistroviridae, Alphatetraviridae, Alphanodavirus, Iridoviridae, Entomopoxvirinae, Baculoviridae, Densovirinae* and *Iflaviridae*. Detection of these viral sequences may be due to insectivorous nature of ducks. The majority of insect virus-like sequences were novel, shared protein similarity of less than 70 % with annotated insect viral proteins. Viral sequences similar to those of cricket paralysis virus, drosophila C virus and pariacoto virus were very abundant in duck gut virome.

Sequences with similarities to plant and fungal viral families (Fig. 2), including *Geminiviridae, Partitiviridae, Tombusviridae, Virgaviridae* and *Secoviridae* were detected in the virome, reflecting dietary habit of ducks. Similarly plant viruses were previously identified in gut virome of pigeon, duck, bat, mouse and human [10, 14, 18, 22, 24, 25]. The duck gut virome contained a large diversity of phage sequences, including members from *Microviridae, Myoviridae, Podoviridae,* and *Siphoviridae* family and unclassified phages. The presence of phages reflected the bacterial flora harbored by ducks in the gastrointestinal tract. Sequences relating to the phages comprise a large proportion (7.33 %) within the duck gut virome. This result is consistent with previous studies, where phage comprise a large fraction of the virome of human, mosquito, pig, ferret, equine and bat faecal samples [13, 14, 18, 26–28]. In summary, the duck gut virome contained a high diversity of viruses of plant, insect and phages in gastrointestinal tract, which may reflect the diet and social habitation of ducks.

### Animal viruses

**Fig. 3:**
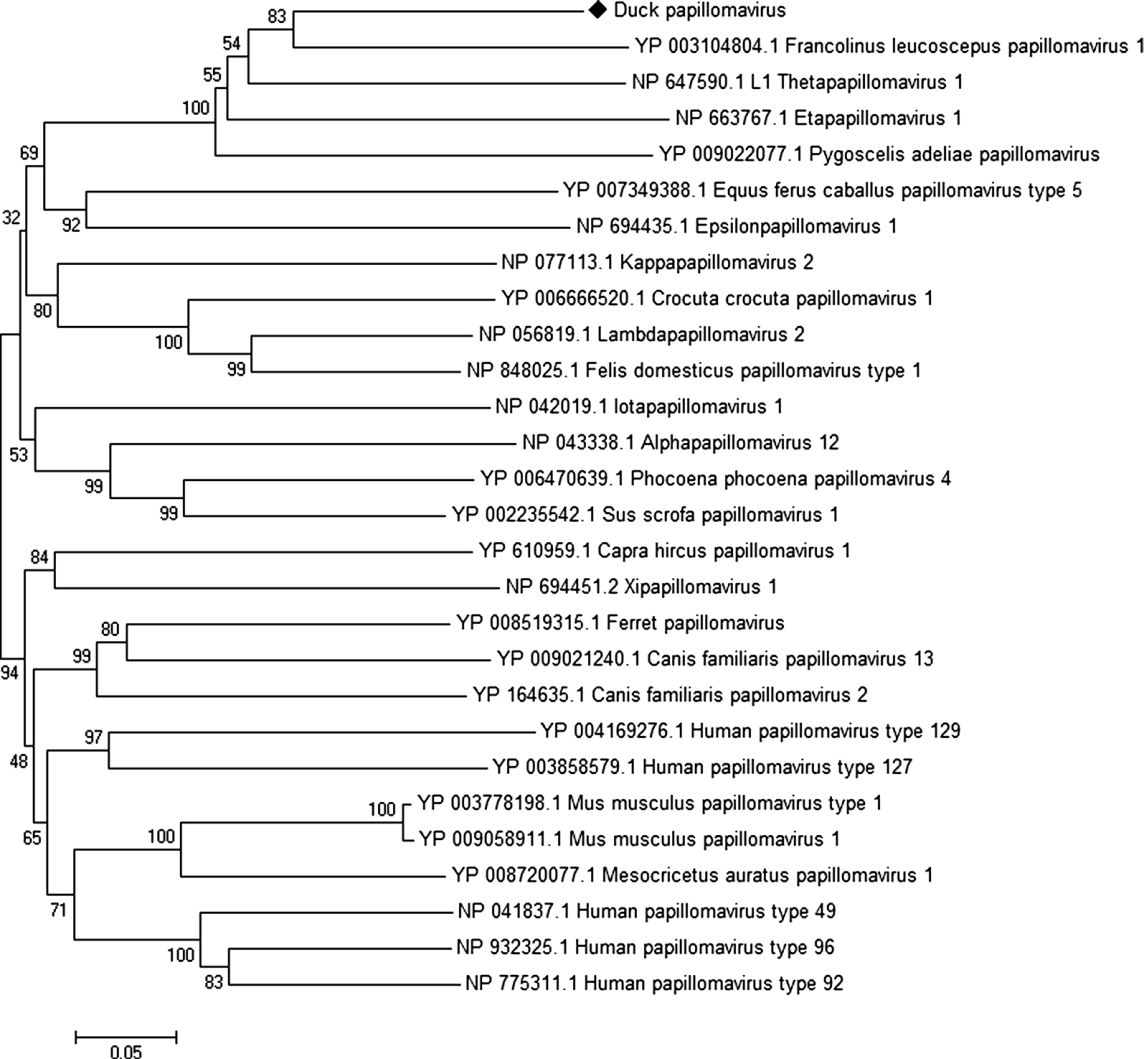
Phylogenetic analysis of translated partial amino acid sequences of L1 gene (1316 nt) of duck papillomavirus with other representative papillomavirus species. The duck-specific sequence identified in duck gut is indicated with a *black diamond*

**Fig. 4:**
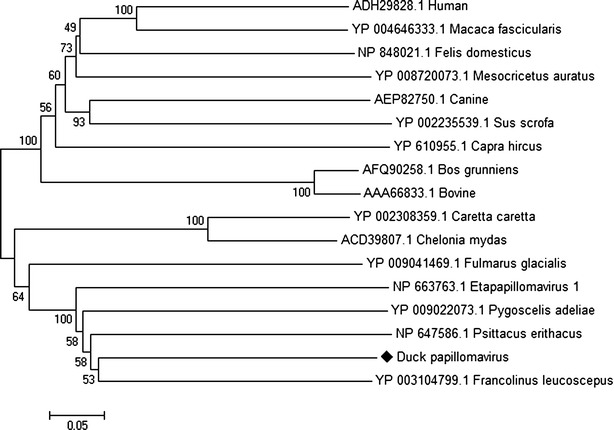
Phylogenetic analysis of translated partial amino acid sequences of E1 gene (4241 nt) of duck papillomavirus with other representative papillomavirus species. The duck-specific sequence identified in duck gut is indicated with a *black diamond*

**Fig. 5:**
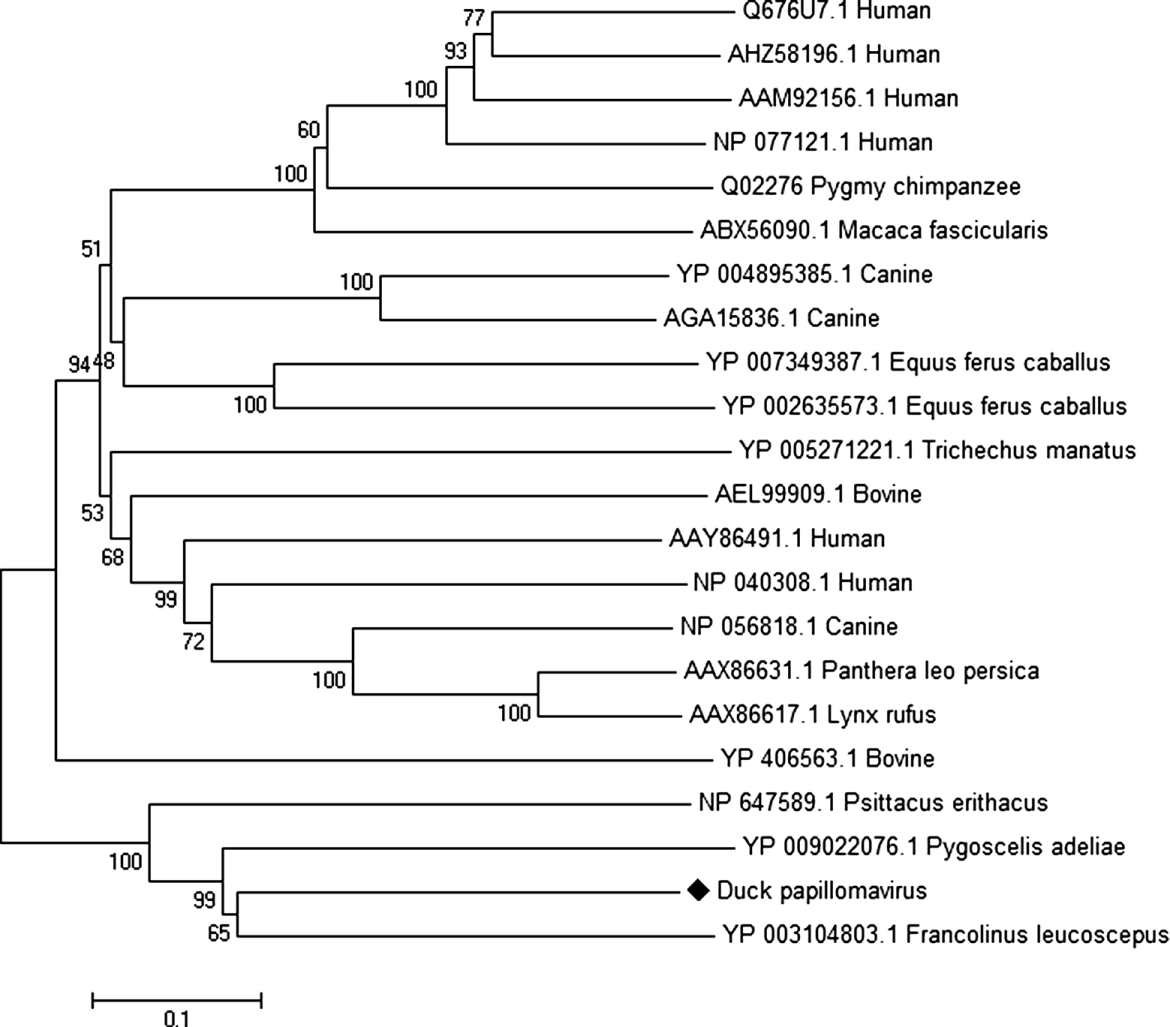
Phylogenetic analysis of translated partial amino acid sequences of L2 gene (1997 nt) of duck papillomavirus with other representative papillomavirus species. The duck-specific sequence identified in duck gut is indicated with a *black diamond*

Papillomaviruses (PVs) are a highly diverse family of small nonenveloped viruses with double stranded circular DNA genomes of 8 kb in size. They infect a wide variety of mammals, as well as birds and reptiles, and are highly species specific and rarely transmitted between species [29, 30]. The circular genome of PVs comprises of a long control region (LCR), the early genes (E1, E2, E4, E6 and E7) and the late genes (L1 and L2) [29, 30]. Among these, the LCR, the replicative proteins E1 and E2 and the capsid proteins L1 and L2 are strictly conserved in all PVs [31]. The current genus classification for papillomavirus types within a genus share more than 60 % nucleotide identity in L1 ORF [30]. Sequences related to members of PVs family were identified in the duck gut virome. BLASTx searches of the duck gut virome sequences showed similarity to various early and late protein sequences of *Francolinus leucoscepus* papillomavirus 1. Phylogenetic analysis of the L1 gene (1316 nt) of putative duck-origin papillomavirus was performed. Duck origin papillomavirus shared the same root with *Francolinus leucoscepus* papillomavirus 1 in the *Dyoepsilonpapillomavirus* genus (Fig. 3). The L1 gene of duck origin papillomavirus sequence shared 72 % nucleotide sequence identities and 68 % amino acid sequence identities with the L1 gene of *Francolinus leucoscepus* papillomavirus 1. In addition, other E1 (4241 nt) and L2 (1997 nt) duck origin papillomavirus sequences also had highest amino acid identity and shared the same root with *Francolinus leucoscepus* papillomavirus 1 (Figs. 4, 5). Previous studies characterized the avian PVs, including *Psittacus erithacus* PV [32], *Fringilla coelebs* PV [33] and *Francolinus leucoscepus* PV type 1 [34]. Papillomaviruses are not typically detected in faecal material. However, recently a full-length papillomavirus genome was characterized from faecal material of a deer mouse [15] and ferrets [18]. Further routine virological approaches, such as viral isolation, complete genome sequencing and other standard approaches will be required to determine the role of duck origin papillomavirus in enteric tract of ducks.

Several contigs and singletons of duck gut virome sequences had significant E scores to the animal viruses. The virome dataset had similarity hits to the duck viral sequences of mallard duck circovirus and anatid herpesvirus 1. Further many sequences also had similarity hits to the avian viral protein sequences, such as fowlpox virus, canarypox virus, pigeonpox virus, *Psittacine* adenovirus 3, chicken picornavirus 2 and raven circovirus. BLASTx analysis of duck virome dataset showed that, three singleton reads had similarity with capsid protein of adeno-associated viruses, one contig sequence related to capsid protein of canine minute virus and one contig related with capsid protein of LuIII virus of *Parvoviridae* family. Similarly duck virome sequences had similarity hits with polyprotein of *Picornaviridae* family members such as mosavirus A2, cosavirus and equine rhinitis A virus. Further, numerous duck gut virome sequences had similarities to the viruses of the families, namely *Circoviridae, Retroviridae* and *Herpesviridae*. However, these sequences are small and cover short portions of the viral genome, so it is not possible to fully characterize these virus related sequences.

## Conclusions

Our study provides a preliminary view of viral diversity present in the virome of the ducks. Due to the short reads, the low quantity of viral load in the sample, or a lack of known reference sequences, we obtained only partial genome sequences in the duck gut virome dataset. Similar results are obtained for bat virome [13, 22, 26]. However, full genome sequences are required to validate these findings. Further, we couldn’t detect any influenza virus related sequences in our duck gut virome dataset. Future studies with deeper sequencing methods and sampling from numerous different locations are required to improve the understanding of the diversity of viruses present in the enteric tract of this aquatic bird.

## Abbreviations

HA: hemagglutinin
NA: neuraminidase
HBSS: hank balanced salt solution
NCBI: national center for biotechnology information
ORF: open reading frame
PVs: papillomaviruses
nt: nucleotide

## References

[1] Huang Y, Li Y, Burt DW, Chen H, Zhang Y (2013). The duck genome and transcriptome provide insight into an avian influenza virus reservoir species. Nat Genet.

[2] Olsen B, Munster VJ, Wallensten A, Waldenström J, Osterhaus AD, Fouchier RA (2006). Global patterns of influenza A virus in wild birds. Science.

[3] Wilcox BR, Knutsen GA, Berdeen J, Goekjian V, Poulson R (2011). Influenza-A viruses in ducks in Northwestern Minnesota: fine scale spatial and temporal variation in prevalence and subtype diversity. PLos One.

[4] Webster RG, Bean WJ, Gorman OT, Chambers TM, Kawaoka Y (1992). Evolution and ecology of influenza A viruses. Microbiol Rev.

[5] Hurt AC, Hansbro PM, Selleck P, Olsen B, Minton C, Hampson AW, Barr IG (2006). Isolation of avian influenza viruses from two different transhemispheric migratory shorebird species in Australia. Arch Virol.

[6] Cardona CJ, Xing Z, Sandrock CE, Davis CE (2009). Avian influenza in birds and mammals. Comp Immunol Microbiol Infect Dis.

[7] Dugan VG, Chen R, Spiro DJ, Sengamalay N, Zaborsky J (2008). The evolutionary genetics and emergence of avian influenza viruses in wild birds. PLoS Pathog.

[8] Taubenberger JK, Kash JC (2010). Influenza virus evolution, host adaptation, and pandemic formation. Cell Host Microbe.

[9] He B, Li Z, Yang F, Zheng J, Feng Y (2013). Virome profiling of bats from myanmar by metagenomic analysis of tissue samples reveals more novel mammalian viruses. PLoS One.

[10] Day JM, Ballard LL, Duke MV, Scheffler BE, Zsak L (2010). Metagenomic analysis of the turkey gut RNA virus community. Virol J.

[11] Edwards RA, Rohwer F (2005). Viral metagenomics. Nat Rev Microbiol.

[12] Tang P, Chiu C (2010). Metagenomics for the discovery of novel human viruses. Future Microbiol.

[13] Li L, Victoria JG, Wang C, Jones M, Fellers GM (2010). Bat guano virome: predominance of dietary viruses from insects and plants plus novel mammalian viruses. J Virol.

[14] Shan T, Li L, Simmonds P, Wang C, Moeser A (2011). The fecal virome of pigs on a high-density farm. J Virol.

[15] Phan TG, Kapusinszky B, Wang C, Rose RK, Lipton HL (2011). The fecal viral flora of wild rodents. PLoS Pathog.

[16] Phan TG, Vo NP, Boros A, Pankovics P, Reuter G (2013). The viruses of wild pigeon droppings. PLoS One.

[17] Chen GQ, Zhuang QY, Wang KC, Liu S, Shao JZ (2013). Identification and survey of a novel avian coronavirus in ducks. PLoS One.

[18] Smits SL, Raj VS, Oduber MD, Schapendonk CME, Bodewes R (2013). Metagenomic analysis of the ferret fecal viral flora. PLoS One.

[19] Larkin MA, Blackshields G, Brown NP, Chenna R, McGettigan PA (2007). Clustal W and clustal X version 2.0. Bioinformatics.

[20] Tamura K, Stecher G, Peterson D, Filipski A, Kumar S (2013). MEGA6: molecular evolutionary genetics analysis version 6.0. Mol Biol Evol.

[21] Ng TF, Marine R, Wang C, Simmonds P, Kapusinszky B (2012). High variety of known and new RNA and DNA viruses of diverse origins in untreated sewage. J Virol.

[22] Donaldson EF, Haskew AN, Gates JE, Huynh J, Moore CJ (2010). Metagenomic analysis of the viromes of three North American bat species: viral diversity among different bat species that share a common habitat. J Virol.

[23] Ng TFF, Willner DL, Lim YW, Schmieder R, Chau B (2011). Broad surveys of DNA viral diversity obtained through viral metagenomics of mosquitoes. PLoS One.

[24] Wu Z, Ren X, Yang L, Hu Y, Yang J (2012). Virome analysis for identification of novel mammalian viruses in bat species from Chinese provinces. J Virol.

[25] Zhang T, Breitbart M, Lee WH, Run JQ, Wei CL (2006). RNA viral community in human feces: prevalence of plant pathogenic viruses. PLoS Biol.

[26] Ge X, Li Y, Yang X, Zhang H, Zhou P (2012). Metagenomic analysis of viruses from bat fecal samples reveals many novel viruses in insectivorous bats in China. J Virol.

[27] Breitbart M, Hewson I, Felts B, Mahaffy JM, Nulton J (2003). Metagenomic analyses of an uncultured viral community from human feces. J Bacteriol.

[28] Cann AJ, Fandrich SE, Heaphy S (2005). Analysis of the virus population present in equine faeces indicates the presence of hundreds of uncharacterized virus genomes. Virus Genes.

[29] de Villiers EM, Fauquet C, Broker TR, Bernard HU, zur Hausen H (2004). Classification of papillomaviruses. Virology.

[30] Bernard HU, Burk RD, Chen Z, van Doorslaer K, Hausen H (2010). Classification of papillomaviruses (PVs) based on 189 PV types and proposal of taxonomic amendments. Virology.

[31] García-Vallvé S, Alonso A, Bravo IG (2005). Papillomaviruses: different genes have different histories. Trends Microbiol.

[32] Tachezy R, Rector A, Havelkova M, Wollants E, Fiten P (2002). Avian papillomaviruses: the parrot *Psittacus erithacus* papillomavirus (PePV) genome has a unique organization of the early protein region and is phylogenetically related to the chaffinch papillomavirus. BMC Microbiol.

[33] Terai M, DeSalle R, Burk RD (2002). Lack of canonical E6 and E7 open reading frames in bird papillomaviruses: *Fringilla coelebs* papillomavirus and *Psittacus erithacus timneh* papillomavirus. J Virol.

[34] Van Doorslaer K, Sidi AO, Zanier K, Rybin V, Deryckère F, Rector A (2009). Identification of unusual E6 and E7 proteins within avian papillomaviruses: cellular localization, biophysical characterization, and phylogenetic analysis. J Virol.

